# Enteric Neuropathy Can Be Induced by High Fat Diet *In Vivo* and Palmitic Acid Exposure *In Vitro*

**DOI:** 10.1371/journal.pone.0081413

**Published:** 2013-12-03

**Authors:** Ulrikke Voss, Elin Sand, Björn Olde, Eva Ekblad

**Affiliations:** 1 Department of Experimental Medical Science, Lund University, Lund, Sweden; 2 Department of Clinical Science Malmö, Lund University, Malmö, Sweden; 3 Department of Clinical Science Lund, Lund University, Lund, Sweden; University of California, Los Angeles, United States of America

## Abstract

**Objective:**

Obese and/or diabetic patients have elevated levels of free fatty acids and increased susceptibility to gastrointestinal symptoms. Since the enteric nervous system is pivotal in regulating gastrointestinal functions alterations or neuropathy in the enteric neurons are suspected to occur in these conditions. Lipid induced intestinal changes, in particular on enteric neurons, were investigated *in vitro* and *in vivo* using primary cell culture and a high fat diet (HFD) mouse model.

**Design:**

Mice were fed normal or HFD for 6 months. Intestines were analyzed for neuronal numbers, remodeling and lipid accumulation. Co-cultures of myenteric neurons, glia and muscle cells from rat small intestine, were treated with palmitic acid (PA) (0 – 10^−3^ M) and / or oleic acid (OA) (0 – 10^−3^ M), with or without modulators of intracellular lipid metabolism. Analyses were by immunocyto- and histochemistry.

**Results:**

HFD caused substantial loss of myenteric neurons, leaving submucous neurons unaffected, and intramuscular lipid accumulation in ileum and colon. PA exposure *in vitro* resulted in neuronal shrinkage, chromatin condensation and a significant and concentration-dependent decrease in neuronal survival; OA exposure was neuroprotective. Carnitine palmitoyltransferase 1 inhibition, L-carnitine- or alpha lipoic acid supplementation all counteracted PA-induced neuronal loss. PA or OA alone both caused a significant and concentration-dependent loss of muscle cells *in vitro*. Simultaneous exposure of PA and OA promoted survival of muscle cells and increased intramuscular lipid droplet accumulation. PA exposure transformed glia from a stellate to a rounded phenotype but had no effect on their survival.

**Conclusions:**

HFD and PA exposure are detrimental to myenteric neurons. Present results indicate excessive palmitoylcarnitine formation and exhausted L-carnitine stores leading to energy depletion, attenuated acetylcholine synthesis and oxidative stress to be main mechanisms behind PA-induced neuronal loss.High PA exposure is suggested to be a factor in causing diabetic neuropathy and gastrointestinal dysregulation.

## Introduction

Obese and/or diabetic patients face an increased risk of multiple complications and decreased quality of life.[1] In a population based survey more than 75% of diabetic patients report gastrointestinal (GI) symptoms.[2] The basis for optimal GI regulation is the enteric nervous system (ENS) innervating the entire digestive tract, and pivotal in coordinating motility, secretion and blood flow.

A patients metabolic and nutritional status contribute to the development of type 2 diabetes (T2D) and obesity is considered the single largest risk factor.[1] The lipid profile in obese and type 2-diabetic patients is characterized by elevated plasma levels of the saturated free fatty acid (FFA) palmitic acid (PA,16∶0) and the monounsaturated FFA oleic acid (OA,18∶1).[3] Lipotoxicity is induced by prolonged exposures to an excess of FFA, particularly PA. Its hallmarks are lipid accumulation in nonadipose tissues, cellular dysfunction and apoptosis.[4], [5] Lipid-induced ENS neuropathy and subsequent pathophysiological signs of GI dysfunction are scarcely studied.

Several animal models exist that display various aspects of T2D. However, except for a few studies, showing reduced concentrations of regulatory peptides,[6] loss of cholinergic and nitrergic neurons, and altered intestinal transit,[7], [8] there have been few reports about ENS plasticity in metabolic disorders. Notable is that diabetics (BMI>30, HbA1c>7%) display loss of enteric neurons, higher incidence of GI symptoms, and reduced glutathione levels indicating oxidative stress as the key event.[9] These reports support the need for further investigations of obesity and T2D related effects on ENS, and consequent intestinal dysfunction.

The aims of the present study were to investigate long-term effects of high fat diet (HFD) on intestinal remodeling and survival of enteric neurons. Additionally, cellular survival and intracellular signaling pathways after exposure to PA and/or OA in mixed myenteric cultures were studied.

## Methods

### Ethics statement

Procedures were approved by the animal ethics committee, Lund and Malmö, SE. Animals were used in accordance with the European Community Council Directive (2010/63/EU) and the Swedish Animal Welfare Act (SFS 1988:534).

### Animals and tissue preparations

Male and female littermate C57BL/6 mice (n = 21), from in house breeding facilities were used in feeding experiments. Mice aged 1 month were divided into two groups; the normal diet (ND) group continuing on standard diet (Lactamin R36)(n = 8) and the HFD group changing to a diet containing 45% kcal from fat (New Brunswick Research diets D12451, USA) (n = 13) (table 1). After 6 months mice were sacrificed by cervical dislocation, weighted and body composition determined by dual-energy x-ray absorpmetry (PIXImus, GE lunar, USA).[10] The GI tract from cardia to rectum was collected, opened along the mesenteric border, emptied and weighed. Segments of ileum and transverse colon were fixed and processed for cryo sectioning.[11]

**Table 1:** Overview of nutritional content of the high fat diet (HFD) and the normal diet (ND).

Nutritional content	HFD g%	ND g%
Protein	24	18.5
Nitrogen free extract	46.2	55.7
Fat:	24.0	4.0
Saturated	8.7	0.6
Monounsaturated	10.8	0.9
Polyunsaturated	4.4	2.6

HFD, Research diets D12451; ND, Lactamin R36; g%, gram percentage

Female Sprauge-Dawley rats (n = 46, 170–260 g), (Charles River, DE) were used for *in vitro* experimentation. Primary cell cultures of the longitudinal smooth muscle layer with adherent myenteric ganglia from small intestine were prepared as described previously.[12] The resulting mixed cell cultures were first grown 4 days in medium (neurobasal A, containing 10% fetal bovine serum, 0.5 mM L-glutamine, 50 U/mL penicillin and 50 µg/mL streptomycin, all from Life Technologies, SE). Fresh medium containing applicable experimental test agents was then added and incubation for an additional period of 4 days followed. Control wells were cultured in parallel. Cells were fixed and subjected to immunocytochemistry.[12]

### Pharmacological agents and experimental *in vitro* set-ups

Stock solutions of PA, methyl palmitic acid (MPA) and pharmacological agents (table 2) were prepared, aliquoted and stored at −20°C.

**Table 2:** Overview of pharmacological substances and brief descriptions of their functions, concentrations used and sources.

Agent	Description	Cat#	Stock	Solvent	Working concentration	Source
PA	Saturated fatty acid, (16∶0)	P9767	10^−2^ M	H_2_O	10^−3^ – 10^−4^ M	Sigma-Aldrich
OA	Monounsaturated fatty acid (18∶1)	O3008	3.3×10^−3^ M	H_2_O	10^−3^ – 10^−4^ M	“
MPA	Nonmetabolizable PA	P5177	10^−2^ M	H_2_O	10^−3^ – 10^−4^ M	“
GW6471	PPAR inhibitor	M6191	0.1 M	DMSO	10^−7^– 3×10^−6^ M	“
Myriocin	Serine palmitoyltransferase inhibitor, inhibit ceramide formation	M1177	10^−7^ M	H_2_O	10^−10^ – 10^−12^ M	“
Etomoxir	Inhibitor of CPT1	E1905	10^−3^ M	H_2_O	10^−5^ – 10^−7^ M	“
L-carnitine	Acyl and acetyl carrier	C0283	0.5 M	H_2_O	10^−2^ – 10^−4^ M	“
Catalase	Antioxidative (H_2_O_2_) enzyme	C9322	10^4^ U/mL	H_2_O	100–3000 U/mL	“
ALA	Enzymatic co-factor, antioxidant	T5625	0.5 M	DMSO	10^−3^ – 10^−5^ M	“
L-NAME	Inhibits NO synthetases	N5751	1 M	H_2_O	10^−3^ M	“
4PBA	Chemical chaperone, inhibits ER-stress	P21005	0.5 M	Methanol	10^−2^ – 5×10^−5^ M	“
Ketamine	NMDA receptor inhibitor	150094	4.2×10^−2^ M	H_2_O	10^−3^ – 3×10^−5^ M	Pfizer, SE
AICAR	AMP analog, activates AMPK	A611-700	0.1 M	H_2_O	3×10^−2^ – 10^−3^ M	Toronto ResearchChemical Inc, CA

PA, palmitic acid; OA, oleic acid; MPA, methyl palmitic acid; ALA, alpha lipoic acid; L-NAME, N-nitro-L-arginine methyl ester; 4PBA, 4-phenyl-butyric acid; AICAR, 5 amino-beta-D-ribofuranosyl-imidazole-4carboxamide; PPAR, peroxisome proliferator-activated receptor; CPT1, carnitine palmitoyl transferase 1; H_2_O_2_, hydrogen peroxide; NO, nitric oxide; NMDA, N-methyl d-aspartate; AMP, adenosine monophosphate; AMPK, AMP activated kinase

PA and MPA were conjugated to BSA (fraction V, Merck, SE) by adding PA- or MPA stock together with 20% BSA w/v to medium, and mixed at 37°C, 1 h prior to use. Final PA:BSA and MPA:BSA molar ratios were 4–5∶1. Final BSA concentration was ≤2% v/v. OA was purchased pre-conjugated to 2% BSA. Different concentrations (10^−4^ – 10^−3^ M) of albuminated FA were added to cultures, controls received medium containing 2% BSA.

Various sets of experiments were performed. 1. Cultures were exposed to medium containing either MPA (10^−4^ – 10^−3^ M), PA (10^−4^– 10^−3^ M), OA (10^−4^– 10^−3^ M) or PA (4×10^−4^ M)+OA (10^−3^ M). 2. Cultures were exposed to control or PA (4×10^−4^ M) enriched medium with or without pharmacological agents (see table 2 for details). 3. Cultures were exposed to medium containing OA (10^−3^ M) or PA (4×10^−4^ M)+OA (10^−3^ M) and enriched with GW6471. 4. Cultures were exposed to either OA (10^−3^ M) or alpha lipoic acid (ALA) (10^−3^ M) enriched medium with or without 5-amino-β-D-ribofuranosyl-imidazole-4-carboxamide (AICAR) (10^−3^ M). 5. Cultures were exposed to AICAR (10^−3^ M) enriched medium with either etomoxir (10^−5^ M) or OA (10^−3^ M)+GW6471 (3×10^−6^ M). Controls were run in parallel.

### Immunocytochemistry and histochemistry

For details on primary and secondary antibodies see table 3. All antibodies were diluted in phosphate buffered saline containing 0.25% Triton X-100 and 0.25% BSA (PBS-T-B). For visualization and quantification of submucous and myenteric neurons, cryosections from ND and HFD mice were immunolabeled with biotin-conjugated HuC/HuD antibodies and vectastain ABC kit (Vector laboratories Inc., USA), according to manufacturer's protocol. Morphometric analyses of small and large intestine were on toludine blue (0.1% in 60% ethanol) stained cryosections. Intracellular lipid accumulations were visualized using Bodipy® 493/503 (Life Technologies, SE) diluted 1:1000 in PBS-T-B, incubated 1 h, washed in PBS-T and mounted in ProLong®gold (Life Technologies, SE).

**Table 3:** Overview of primary and secondary antibodies used in immunocytochemistry.

Raised against	Dilution	Code	Source	Host	References
Human neuronal protein, (HuC/HuD)	1∶600	A21272	Life Technologies, SE	Mouse	[12], [13]
Human gene product 9.5, (PGP 9.5), purified human brain	1∶1.200	RA95101	Ultraclone, UK	Rabbit	[13]
S100A and B (S100), purified bovine brain	1∶12.000	Z0311	DAKO, DK	Rabbit	[14]
Alpha smooth muscle actin (aSMA), synthetic N-terminal	1∶600	A2547	Sigma-Aldrich, SE	Mouse	[15]
Dy-light 488 anti-mouse IgG	1∶1.000	115-485-166	Jackson Lab Inc, USA	Goat	
Dy-light 594 anti-mouse IgG	1∶1.000	715-515-151	Jackson Lab Inc, USA	Donkey	
Dy-light 594 anti- rabbit IgG	1∶1.000	711-515-152	Jackson Lab Inc, USA	Donkey	

Double immunolabeling of cultures were by overnight incubation in moist chamber at 4°C with a mixture of primary antibodies. Secondary antibodies were mixed and incubated 1 h at RT. Hoechst (Life Technologies, SE) cell nuclei counter staining was performed according to manufacturer's protocol. Intracellularly accumulated lipid droplets were visualized with Bodipy® 493/503 added to secondary antibodies at final dilution of 1∶1000. Mounting was in PBS∶glycerol 1∶1 followed by fluorescence microscopy (Olympus BX43, LRI, SE) with appropriate filter setting.

### EdU incorporation

Proliferating cells were identified by Click-iT EdU Alexa flour 488 imaging kit (Life Technologies, SE), according to manufacturer's protocol. EdU was added to culture wells 24 h prior to fixation.

### Morphometic analyses and cell calculations

Small and large intestinal mucosa and muscularis propia thicknesses were estimated for each mouse using the mean from ten representative measurements (Aperio ScanScope CS/GL SS5082 and ImageScope). The numbers of HuC/HuD-immunoreactive submucous and myenteric neuronal cell bodies were counted per mm section length. From each mouse, cryosections (10 µm) cut longitudinally at 3–4 different depths comprising a total length of 25–30 mm, were used.

Neuronal survival *in vitro*, after exposure to the various treatments, was calculated by counting the total number of surviving neurons in the entire culture well (69 mm^2^) and expressed as percentage of the number of neurons in the control well run in parallel. Survival of glia, muscle cells and proliferating EdU positive cells were, due to their abundance, calculated by capturing (Olympus cellSens) five randomized microscopic fields (center and each quadrant, 0.45 µm^2^ per field) from every culture well (n = 3–38). From these selections the total cell number, using Hoechst staining, and the total number of either S100 or alpha smooth muscle actin (aSMA) immunoreactive (expressed as percentage of control) or EdU positive cells (expressed as percentage of total) were estimated. Calculations were done using Image J cell calculator (rsb.info.nih.gov/ij/). Measurements on soma and nuclei area, cellular perimeter as well as on neuronal chromatin condensation were done using the Olympus cellSens system. Twenty cells of each type were randomly identified by immunocytochemistry in each culture well and analyzed, n = 5 in each group.

### Statistical analyses

Data are presented as means ± SEM and analyzed by GraphPad Prism (GraphPad Software Inc, USA). Statistical significances were determined using two-way-analysis-of-variance, followed by Bonferronis post hoc test (*in vivo* data), or one-way-analysis-of-variance followed by Dunnet's post hoc test towards controls (*in vitro* data). A confidence level of 95% was considered significant.

### Data availability

We agree to make freely available any materials and data described in the publication that may be reasonably requested for the purpose of academic, non-commercial research.

## Results

### HFD compared to ND

**Figure 1:**
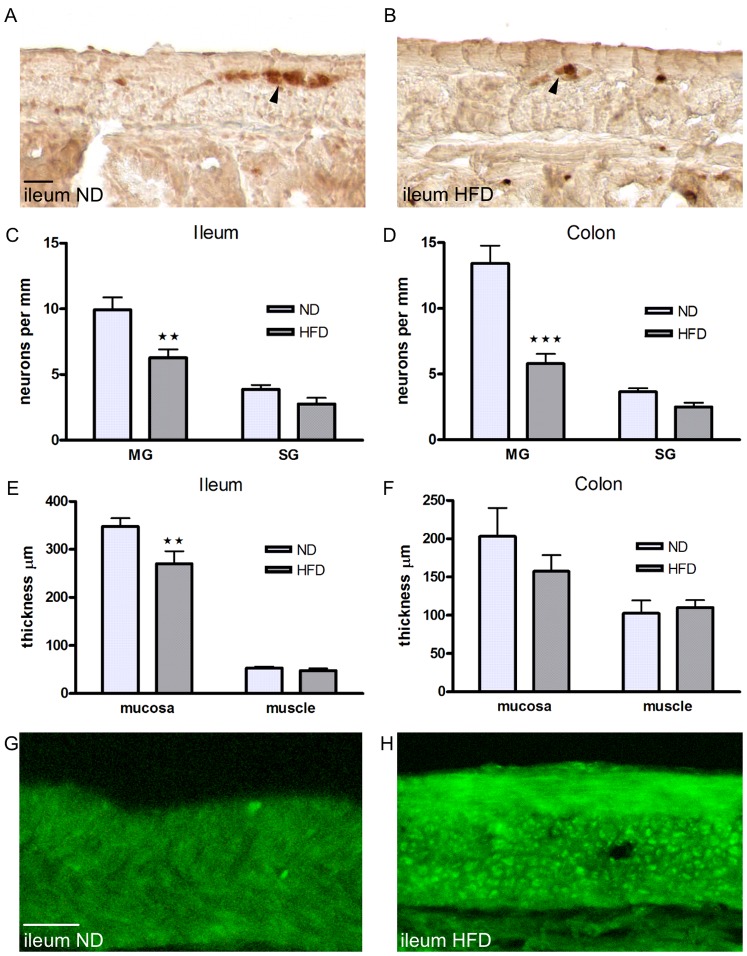
Effects of HFD on intestinal morphology. Representative micrographs (A, B, G, H), numbers of neurons in myenteric (MG) and submucous ganglia (SG) (C, D), and morphometrics (E, F) in ileum and colon from mice fed a high fat diet (HFD, dark grey bars) or a normal diet (ND, light grey bars) for 6 months. A–B cryosections from ileum of ND (A) and HFD (B) immunostained for HuC/HuD-biotin. Myenteric neurons, are indicated with arrowheads. C–D numbers of neurons per mm in longitudinally cut sections of the intestine. HFD reduces myenteric neuronal density in ileum (C) and colon (D) compared to ND. Numbers of submucous neurons are unchanged in both intestinal regions. E–F HFD causes a reduction of mucosal thickness in ileum (E), but not in colon (F) compared to ND. G–H cryosections from ileum of ND (G) and HFD (H) mice, stained with Bodipy®493/503 to illustrate lipid droplets within muscularis propria. HFD markedly increases intramuscular lipid droplet formation. Data (C–F) are expressed as mean ± SEM, ** p<0.01, *** p<0.001, in C–D n = 8 ND, n = 13 HFD, in E–F n = 5 ND, n = 6 HFD. Bars represent 20 µm.

Mice fed HFD displayed increased body weight (p<0.001) and body fat percentage (p<0.001) compared to ND mice. Weights of the GI tract did not differ between the groups (ND 3.3 g±0.3, HFD 3.6 g±0.3). In mice fed HFD, compared to littermates on a ND, there were significant reductions in the numbers of myenteric neuronal cell bodies in both ileum and colon (ileum p<0.01, colon p<0.001), on the other hand the numbers of both ileal and colonic submucous nerve cell bodies were unchanged. In ileum of HFD mice, the mucosal thickness was reduced compared to ND mice (p<0.01), while no difference was noted in the muscularis propria. Colon mucosa and muscularis propria thicknesses were identical in both groups. In both ileum and colon from mice fed HFD, intramuscular lipid accumulations were numerous and more prominent, compared to mice fed a ND. Results are illustrated and summarized in figure 1.

### Survival of myenteric neurons, smooth muscle cells and glia *in vitro*

Primary cultures of the outer muscle layer with adherent myenteric ganglia from rat small intestine include neurons, glia and intestinal smooth muscle cells. All experimentations comprised 4 days recovery culture period, followed by 4 days of treatment. Controls were always run in parallel and cellular survival estimated as percentage of control.

#### Effects of PA, OA and MPA on cellular survival

**Figure 2:**
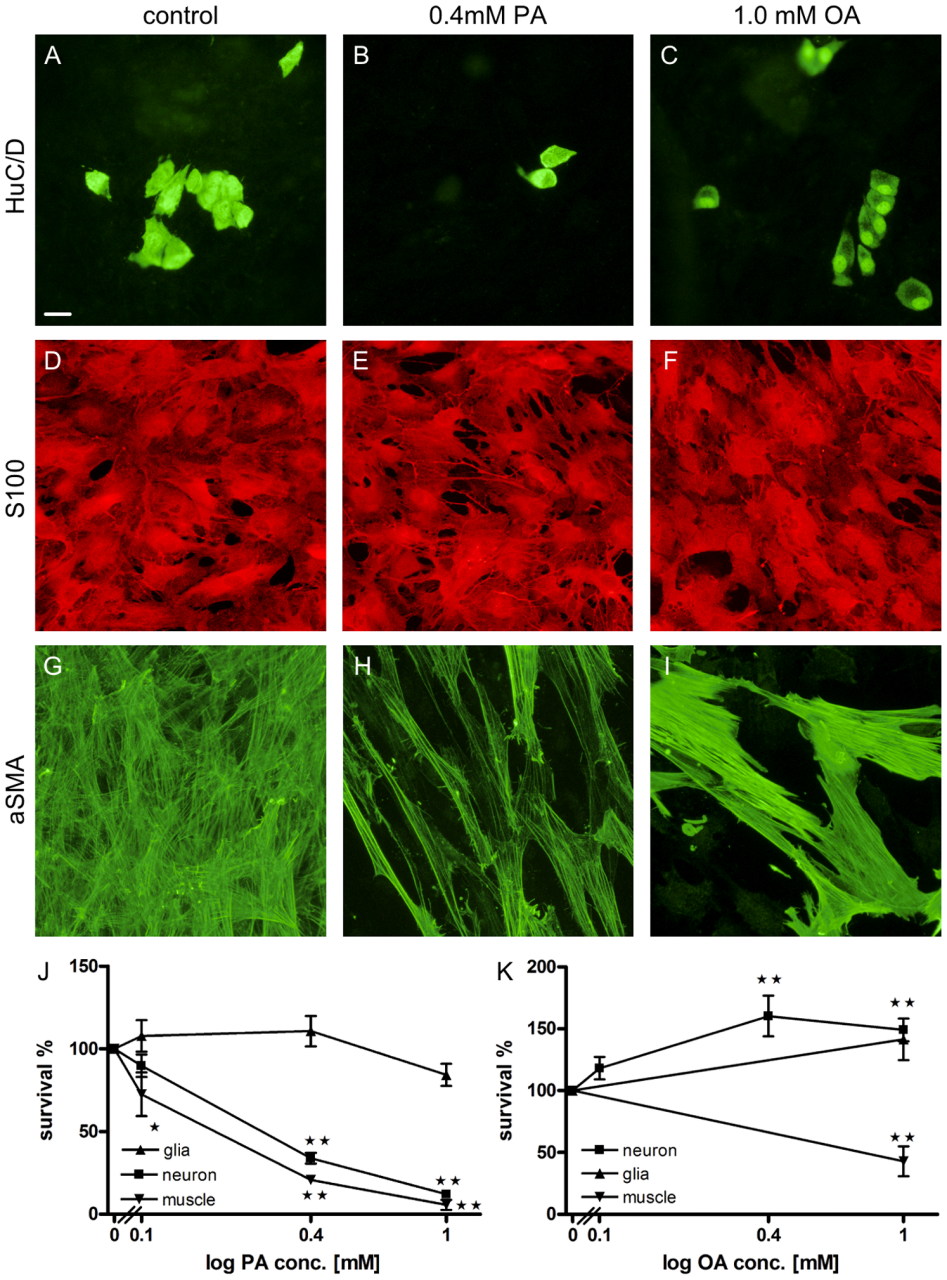
PA and OA have different effects on cellular survival *in vitro*. Effects of control, PA or OA treatments on neurons, glia and muscle cells *in vitro*. A–I representative micrographs of neurons (A–C), glia (D–F) and muscle cells (G–I) after control (A, D, G), PA (4×10^−4^ M) (B, E, H) or OA (10^−3^ M) (C, F, I). Survival of neurons (▪) glia (▴) and muscle cells (▾) *in vitro* after 4 days treatment with PA (10^−4^ – 10^−3^ M) (J) or OA (10^−4^ – 10^−3^ M) (K). PA causes a severe and concentration-dependent loss of both neurons and muscle cells, but does not change survival of glia. OA is neuroprotective and causes a concentration-dependent increase in survival of neurons. Presence of OA does not change numbers of glia, but severely reduces survival of muscle cells. Data presented as mean ± SEM, n = 5–41, * p<0.05, ** p<0.01. Bar represents 20 µm.

Survival after exposure to PA (10^−4^ – 10^−3^ M), OA (10^−4^ – 10^−3^ M) or PA (4×10^−4^ M)+OA (10^−3^ M) were determined. Control wells displayed a large number (4.3±0.2/mm^2^, n = 43) of evenly distributed neuronal cell bodies with rich terminal networks. Addition of PA severely reduced, while OA increased, the number of surviving neurons in a concentration-dependent manner. Addition of PA+OA resulted in neuronal survival similar to control. Numerous glia was present in control (355±30.5/mm^2^, n = 24) and no changes were observed after PA and/or OA treatments. In control wells muscle cells were frequent (795±72/mm^2^, n = 22) and provided a coalescent layer. After PA or OA treatment muscle cells disappeared in a concentration-dependent manner. Notable was that the combined treatment of PA+OA resulted in an increased survival of muscle cells compared to control. Figure 2 and table 4 illustrate and summarize findings on PA and/or OA treatment.

**Table 4:** Effects of test agents on PA and OA and AICAR treated cultures.

Treatment	Neuron	Glia	Muscle
Control medium	100	100	100
**PA** 4×10^−4^ M	38.1±2.0 **	**110.8±9.2**	20.9±2.3 **
**OA** 10^−3^ M	165.5±11.4**	**114.5±29.9**	43.0±12.1**
4×10^−4^ M PA+OA			
10^−4^ M	96.0±5.5	-	-
4×10^−4^ M	119.6±14.9	-	-
10^−3^ M	123.5±20.9	92.4±20.4	133.0±9.5 **
OA+GW6471			
10^−7^ M	117.7±6.7	-	-
10^−6^ M	125.2±19.9	-	-
3×10^−6^ M	127.8±12.7	95.3±8.5	44.7±18.7 **
4×10^−4^ M PA+OA+GW6471			
10^−6^ M	103.3±15.5	-	-
3×10^−6^ M	94.7±20.7	109.0±32.7	91.7±6.8
4×10^−4^ M PA+myriosin			
10^−12^ M	43.2±5.3 **	-	-
10^−11^ M	54.7±9.5 **	-	-
10^−10^ M	40.7±3.8 **	144.8±35.7	9.7±3.0 **
4×10^−4^ M PA+etomoxir			
10^−7^ M	68.5±12.8	-	-
10^−6^ M	61.0±6.1 *	-	-
10^−5^ M	84.0±13.5	164.0±40.2	11.1±4.7 **
4×10^−4^ M PA+carnitine			
10^−4^ M	56.2±8.8 *	-	-
10^−3^ M	59.2± 6.9 *	-	-
10^−2^ M	92.3±8.8	75.7±0.5	27.0±13.0 **
4×10^−4^ M PA+catalase			
100 U/mL	51.6±6.4 **	-	-
1000 U/mL	47.0±5.3 **	81.7±91.1	30.4±10.1 **
3000 U/mL	56.7±6.9 **	-	-
4×10^−4^ M PA+ALA			
10^−5^ M	68.0±12.6 *	-	-
10^−4^ M	84.0±5.7	-	-
10^−3^ M	90.3±8.4	132.0±28.7	7.9±3.7 **
4×10^−4^ M PA+L-NAME			
10^−4^ M	54.5±12	-	-
4×10^−4^ M PA+4PBA			
5×10^−4^ M	64.1±5.1 **	-	-
5×10^−3^ M	63.9±8.1 **	-	-
10^−2^ M	54.0±9.5 **	112.1±8.6	36.1±7.3 **
4×10^−4^ M PA+ketamine			
3×10^−5^ M	55.8±9.7 **	-	-
10^−4^ M	60.0±5.6 **	-	-
10^−3^ M	50.0±5.8 **	66.0±24.9	1.7±1.1 **
AICAR			
10^−4^ M	67.5±13.4**	-	-
10^−3^ M	50.4±6.7**	-	-
3×10^−3^ M	38.8±14.8**	103.5±19.5	69.0±11.7
4×10^−4^ M PA+AICAR			
10^−4^ M	49.8±3.8**	-	-
10^−3^ M	29.3±5.5**	-	-
3×10^−3^ M	14.3±7.0**	94.1±8.1	48.5±13.5 **
10^−3^ M AICAR+etomoxir			
10^−5^ M	49.7±3.6 **	101.1±21.6	83.9±13.2
10^−3^ M AICAR+OA			
10^−3^ M	89.6±10.6	105.8±23.3	30.2±8.7 **
10^−3^ M AICAR+ALA			
10^−3^ M	63.7±10.3 **	109.5±37.5	23.3±3.8 **
10^−3^ M AICAR+10^−3^ M OA+GW6471			
3×10^−6^ M	51.3±3.7 **	72.4±11.2	14.7±8.9 **

Survival of cultured neurons, glia and intestinal muscle cells, after 4 days recovery period followed by 4 days in control medium, PA (4×10^−4^ M), OA (10^−3^ M), PA (4×10^−4^ M) + test agents, OA (10^−3^ M) + test agents or AICAR (10^−3^ M) + test agents. Data is presented as percentage of control and given as mean ± SEM, n = 3–38, *p<0.05, ** p<0.01 as compared to control, (–) not determined. To facilitate comparisons, data on cellular survival from control, PA and OA are highlighted in bold.

MPA is a nonmetabolizable form of PA. Neuronal survival in cultures treated with MPA (10^−4^ – 10^−3^ M) did not differ from controls (table 5). All wells displayed evenly distributed nerve cell bodies, with terminal networks, without signs of morphological alterations. Also muscle cells and glia were unaffected in appearance and numbers after MPA treatment (table 5).

**Table 5:** Effects of pharmacological test agents on cultures.

Treatment	Neuron	Glia	Muscle
Control medium	100	100	100
MPA			
10^−4^ M	99.4±6.9	88.5±9.6	87.0±30.0
4×10^−4^ M	99.1±7.6	68.0±16.7	84.5±17.5
10^−3^ M	111.9±5.8	75.5±9.9	103.0±27.0
GW6471			
10^−7^ M	95.3±6.7	-	-
10^−6^ M	107.7±9.8	-	-
3×10^−6^ M	130.0±32.8	88.8±6.7	113.3±34.3
Myriosin			
10^−12^ M	105.2±5.2	-	-
10^−11^ M	107.4±14.2	-	-
10^−10^ M	99.5±8.2	122.4±29.4	66.5±7.1**
Etomoxir			
10^−7^ M	100.9±5.4	-	-
10^−6^ M	93.1±5.6	-	-
10^−5^ M	104±6.7	92.5±20.4	110.7±35.1
L-Carnitine			
10^−4^ M	113.7±13.2	-	-
10^−3^ M	138.0±15.2 *	-	-
10^−2^ M	160.8±8.3 **	86.5±2.5	97.5±16.5
Catalase			
10 U/mL	92.83±7.4	-	-
1000 U/mL	85.67±7.5	87.8±3.6	70.8±10.8
3000 U/mL	94.5±17.4	-	-
ALA			
10^−5^ M	125.1±14.5	-	-
10^−4^ M	126.9±11.9	-	-
10^−3^ M	157.3±14.1 *	95.4±16.6	57.4±11.4**
L-NAME			
10^−4^ M	101.0±8.8	-	-
4PBA			
5×10^−4^ M	112.3±4.2	-	-
5×10^−3^ M	100.5±11.5	-	-
10^−2^ M	71.67±34.4	108.2±20.0	94.3±15.8
Ketamine			
3×10^−5^ M	97.2±11.82	-	-
10^−4^ M	93.6±8.0	-	-
10^−3^ M	84.8±4.3	93.7±21.1	74.1±4.1

Survival of cultured neurons, glia and intestinal muscle cells, after 4 days recovery period followed by 4 days in test agent. Control medium is included for comparison. Data is presented as percentage of control and given as mean ± SEM, n = 5-38, * p<0.05, ** p<0.01, (–) not determined.

### PA changes the morphology of neurons and glia

**Figure 3:**
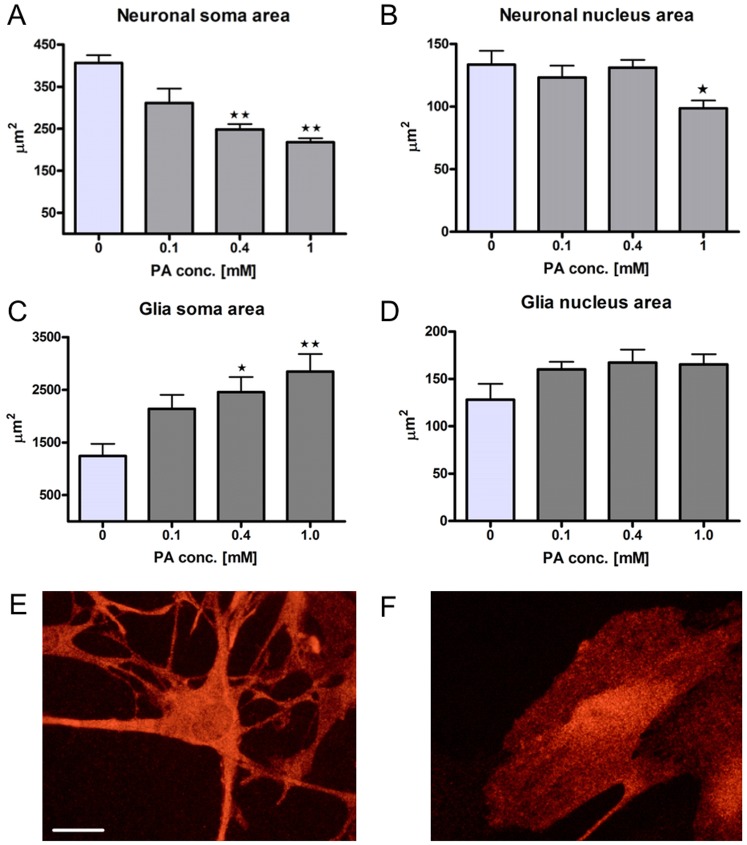
PA changes the morphology of neurons and glia *in vitro*. Morphometric analyses (A–D) of soma and nucleus in cultured neurons (A, B) and glia (C, D) after PA treatment compared to control (0). PA (10^−4^ – 10^−3^ M) treatment significantly and concentration-dependently decreases neuronal soma-area. PA (10^−4^ – 10^−3^ M) treatment significantly and concentration-dependently increases glia soma area. Neuronal nucleus size was unchanged at PA concentrations up to 4×10^−4^ M but was significantly reduced at 10^−3^ M. Glia nucleus size was not changed in any of the tested PA concentrations. E-F, representative micrographs of cultured glia in control (E) and after PA (4×10^−4^ M) treatment (F), depicting the PA-induced transformation of glia, from a stellate to a round phenotype. Data presented as mean ± SEM, n = 5, * p<0.05, ** p<0.01. Bar represents 20 µm.

**Figure 4:**
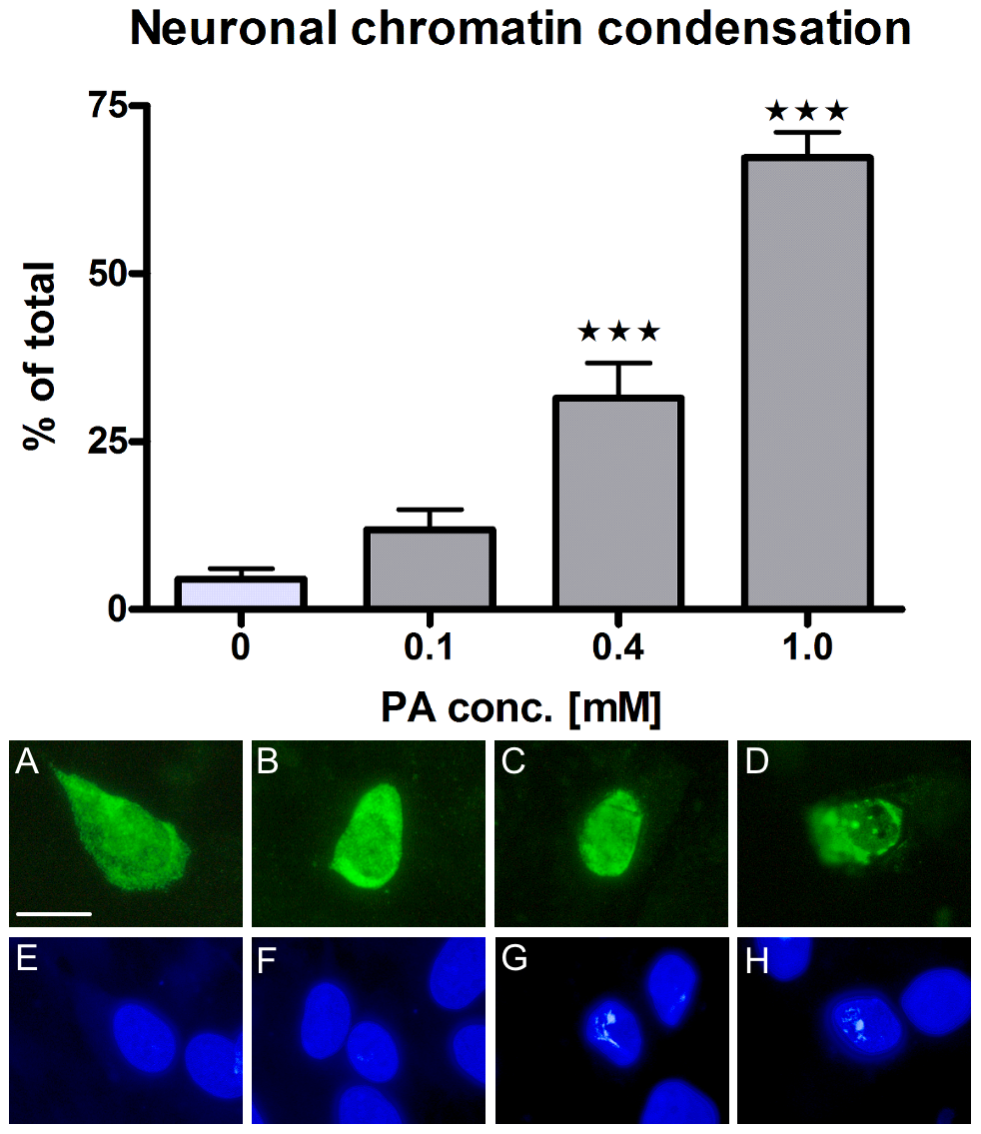
PA causes neuronal chromatin condensation *in vitro*. Presence of neuronal chromatin condensation *in vitro* after PA treatment (10^−4^ – 10^−3^ M) compared to control (0). Upper panel: PA increases the number of neurons displaying nuclear chromatin condensation in a concentration-dependent manner. Lower panel: representative micrographs of HuC/HuD immunolabeled neurons (A–D) and Hoechst labeled nuclei (E–H) after control and PA (10^−4^ M – 10^−3^ M) treatment. Increasing concentrations of PA cause shrinkage of neuronal soma and nuclei and increase chromatin condensation (revealed as white spots in Hoechst stain). (A, E) control, (B, F) PA (10^−4^ M), (C, G) PA (4×10^−4^ M) and (D, H) PA (10^−3^ M). Data shown as mean ± SEM, *** p<0.001, n = 5, Bar represents 20 µm.

Nerve cell bodies displayed a markedly reduced area in response to PA treatment as compared to controls (p<0.01). At 10^−3^ M PA a reduced (p<0.05) nuclei area was observed (figure 3). The percentage of neurons displaying chromatin condensation, in response to PA treatment increased concentration-dependently (figure 4). Although glia perimeter and nucleus size remained unchanged, glia soma-area markedly increased after PA exposure (p<0.01), which was manifested in the transition from a stellate to a rounded phenotype (figure 3 E, F).

### Lipid droplet accumulation after PA and OA treatment

**Figure 5:**
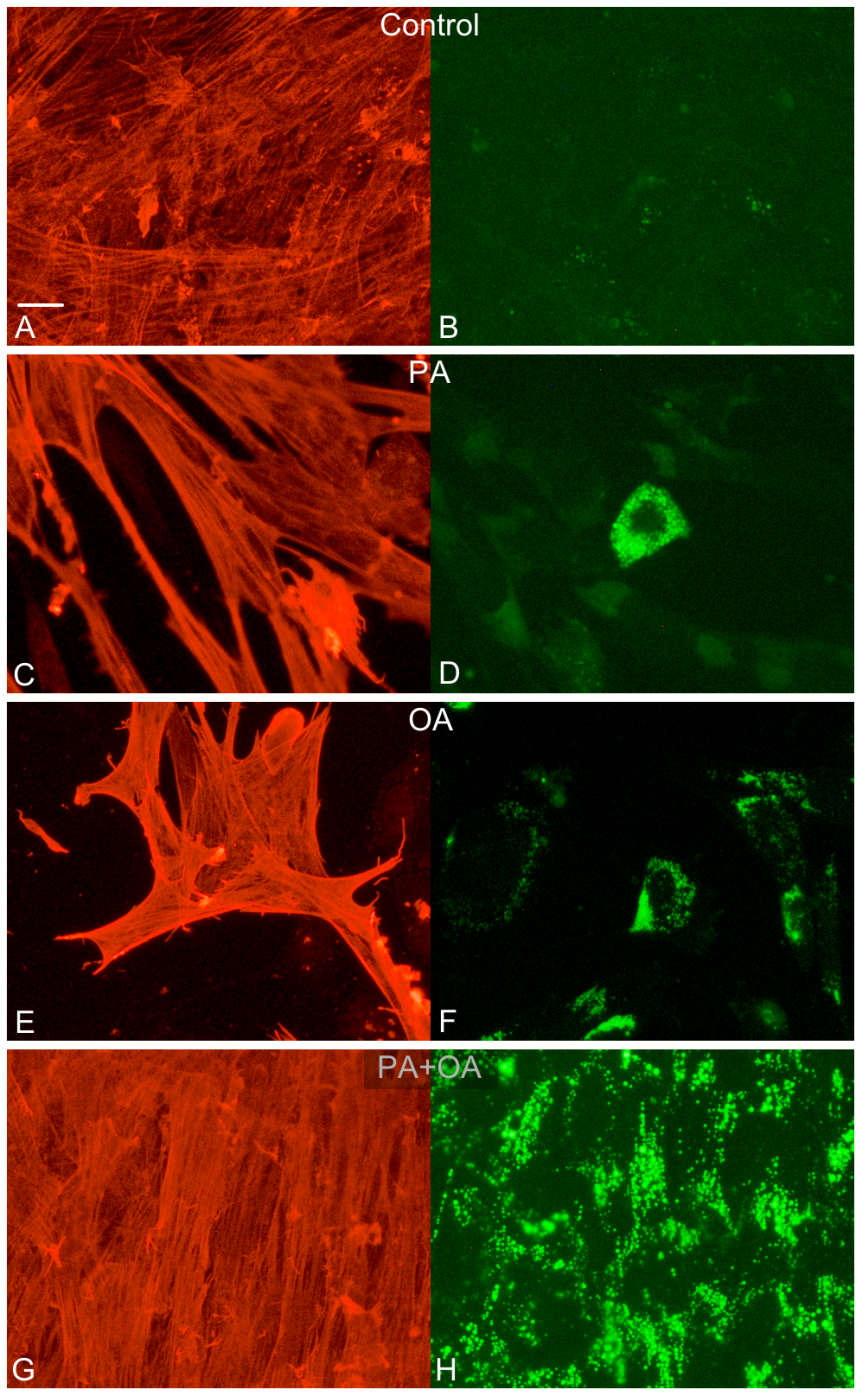
PA and OA affect lipid droplet accumulation in smooth muscle cells in vitro. Representative micrographs of lipid droplet accumulations in vitro in aSMA-immunoreactive (A, C, E, G) and Bodipy® stained (B, D, F, H) muscle cells. The latter for visualization of lipid droplet accumulation. (A, B) control, (C, D) PA (4×10^−4^ M), (E, F) OA (10^−3^ M) and (G, H) PA (4×10^−4^ M) plus OA (10^−3^ M) treatment. Control conditions resulted in low accumulation of lipids (B). Both PA and OA each causes loss of smooth muscle cells (C, E) and a subpopulation of those remaining is densely packed with lipids (D, F). The combined treatment with PA and OA results in a well preserved muscle cell survival and markedly increased lipid droplet accumulation in all muscle cells (H). Bar represents 20 µm.

In control and at all PA and OA concentrations tested Bodipy® staining revealed absence of lipid accumulation in neurons and glia. In controls, a few small lipid droplets were occasionally observed in muscle cells. After exposure to PA (4×10^−4^ M) or OA (10^−3^ M) the numbers of muscle cells were low; those remaining harboured intracellular lipid droplets of varying amount and size. The combined treatment with PA and OA (see above) failed to induce loss of muscle cells, but caused a substantial increase in lipid droplet accumulation within the majority of muscle cells. Lipid droplets were exclusively localized within the cytoplasm. Results are illustrated in figure 5.

### Proliferation after PA and OA exposure

EdU labeling revealed the number of proliferating cells in the cultures; individual cell types were not taken into consideration. Presence of PA (10^−4^–10^−3^ M) or OA (10^−3^ M) reduced the relative frequency of proliferating cells (p<0.01). Proliferation after addition of MPA (10^−4^–10^−3^ M) or PA (4×10^−4^ M)+OA (10^−3^ M) did not differ from control (table 6).

**Table 6:** Relative proliferation of cells in culture.

Treatment	% EDU incoporation
Control medium	55.4±3.9
PA	
10^−4^ M	37.6±9.8
4×10^−4^ M	36.9±3.0 **
10^−3^ M	26.7±0.7 **
OA	
10^−3^ M	25.0±4.9 **
PA+OA	
4×10^−4^ M+10^−3^ M	51.0±3.2
MPA	
10^−4^ M	47,2±4.3
4×10^−4^ M	61.0±3.8
10^−3^ M	56.8±1.2

Relative (% of total) proliferation of cells *in vitro* determined after 24 h EDU incorporation, without considering individual cell types. Treatment with PA (10^−4^ – 10^−3^ M) or OA (10^−3^ M) reduce the relative proliferation compared to control. MPA and the combined treatment of PA (4×10^−4^ M) and OA (10^−3^ M) maintain proliferation compared to control. Data is presented as mean ± SEM, n = 3–4, * p<0.05, ** p<0.01.

### PA and OA effects: cellular signalling and pathway identification

To identify the underlying mechanisms for the evident PA and OA effects *in vitro*, inhibitors of signalling molecules and pathway activators involved in intracellular lipid metabolism were tested. Cultures were incubated in control, PA (4×10^−4^ M) or OA (10^−3^ M) enriched medium with or without pharmacological test agents. Results from these experiments are outlined below and presented in detail in tables 4 and 5.

#### Peroxisome proliferator-activated receptor (PPAR) activation

OA-induced neuroprotection in central neurons involves activation of PPAR alpha.[16] It was tested if this was true for enteric neurons as well. Presence of PPAR antagonist GW6471 (10^−7^– 3×10^−6^ M) did not reverse OA-induced neuroprotection or loss of muscle cells. GW6471 alone did not change cellular survival nor did it affect OA-evoked rescue of PA-induced neuronal and muscle cell losses.

#### Ceramide formation

Inhibiting PA from entering the ceramide pathway by adding serine palmitoyltransferase inhibitor myriocin (10 ^−12^ – 10 ^−10^ M) did not reverse PA-induced neuronal or muscle cell losses. Myriocin alone reduced survival of muscle cells but did not change that of neurons or glia.

#### Palmitoylcarnitine formation

The carnitine palmitoyltransferase 1 (CPT1) inhibitor etomoxir (10 ^−7^ – 10 ^−5^ M) prevents conversion of PA-CoA to palmitoylcarnitine in mitochondrial, peroxisome and endoplasmatic reticulum (ER) membranes. Its presence reversed PA-induced neuronal, but not muscle cell losses in a concentration-dependent manner. Etomoxir alone did not change survival of any of the cultured cell types.

L-carnitine (10^−4^–10^−2^ M) supplementation to cultures concentration-dependently reversed PA-induced neuronal loss. L-carnitine alone was neuroprotective (p<0.01), but had no effect on glia or muscle cell survival, nor did it reverse PA-induced loss of muscle cells.

#### Reactive oxygen species (ROS) and ER stress

Catalase is the main enzyme handling hydrogen peroxide. Presence of catalase (100–3000 U/mL), did not reverse PA-induced neuronal or muscle cell losses. Catalase alone did not change cellular survival. Besides hydrogen peroxide ROS includes peoxynitrite, hydroxyl, peroxyl and hypochlorus radicals as well as singlet oxygen. These were scavenged using the antioxidant, ALA (10 ^−4^–10 ^−3^ M) which concentration-dependently reversed PA-induced neuronal, but not muscle cell losses. ALA alone increased neuronal survival (p<0.05), reduced muscle survival but did not change survival of glia. To test possible contributions of nitric oxide or peroxynitrite on PA-induced neuronal loss N-nitro-L-arginine methyl ester (L-NAME), a general inhibitor of nitric oxide synthetases, was used. L-NAME (10^−3^ M) alone did not change neuronal survival nor did it rescue neurons from PA-induced loss.

Elevated levels of ROS and oxidative stress may cause ER stress. The chaperone 4-phenyl-butyric acid (4PBA) is a stabilizor of protein folding. 4PBA (5×10^−4^–10^−2^ M) did not reverse PA-induced neuronal or muscle cell losses. 4PBA alone did not change cellular survival.

#### Excitotoxicity

PA metabolism may lead to excess formation of glutamate, causing neurotoxicity by hyperactivation of NMDA receptors. Ketamine (3×10^−5^–10^−3^ M), a NMDA receptor blocker, did not reverse PA-induced neuronal or muscle cell losses. Ketamine alone did not change cellular survival.

### AMP activated kinase (AMPK) activation mimics PA exposure

AMPK is a central kinase in metabolism, regulating pathways like beta-oxidation and glucose uptake. The AMP analog AICAR induces AMPK activation. AICAR (10^−3^– 3×10^−2^ M) alone induced a concentration-dependent neuronal loss (p<0.01), in the same range as that caused by PA (4×10^−4^ M). AICAR and PA did not potentiate each other when added simultaneous. Etomoxir (10^−5^ M) did not reverse AICAR-induced neuronal loss. AICAR (3×10^−2^ M) exposure did not change the numbers of glia or muscle cells. OA (10^−3^ M) protected neurons against AICAR (10^−3^ M) -induced neuronal loss while ALA (10^−3^ M) did not. GW6471 (3×10^−6^ M) inhibited OA (10^−3^ M) -mediated reversal of AICAR (10^−3^ M)-induced neuronal loss (table 4).

## Discussion

Long-term intake of HFD caused reduced numbers of myenteric neurons in ileum and colon, a thinner ileal mucosa and lipid accumulation in muscularis propria. The mechanisms by which prolonged exposure to lipids and lipid metabolites cause neuropathy and morphologic remodeling were addressed in the current study.

The dominating FA's in HFD, PA and OA, were tested *in vitro* for their ability to mimic the neuropathy and lipid handling noted *in vivo*. While PA evoked a detrimental effect on neurons OA was clearly neuroprotective. Both PA and OA caused intestinal muscle cell loss, but unchanged glial survival. In addition PA caused shrinkage and chromatin condensation of neurons and transition of glia to a rounded phenotype. Substituting PA for nonmetabolizable MPA did not affect cellular survival. This strongly suggests PA-induced neuronal and muscle cell losses are due to uptake and intracellular handling of PA.

### Mechanisms behind PA-induced neuronal loss

PA-induced neurodegeneration occurs in neuronal cell lines and is suggested to be caused by oxidative and ER stress.[17], [18] In the present study the underlying mechanisms for PA-induced myenteric neuronal loss were elucidated by intervening pharmacologically with several intracellular pathways in lipid metabolism. The results indicate that ceramide and hydrogen peroxide formation as well as glutamate generated excitotoxicity all could be excluded as underlying mechanisms. The studies pointed towards energy depletion, halted acetylcholine synthesis and oxidative stress as major events.

The CPT1 inhibitor etomoxir, L-carnitine and the important enzymatic co-factor and antioxidant ALA,[19] all protected myenteric neurons from PA-induced loss. Although attenuation of PA stimulated beta-oxidation would be expected to result in reduced ROS production, this pathway is less likely as neurons, including ENS, are not considered relying on beta-oxidation for energy production. A condition reflected in low neuronal expression levels of CPT1a and b.[20], [21] On the other hand CPT1c is abundantly expressed and localized to the ER, [21], [22] suggesting biosynthetic rather than energy related functions. CPT1c mediates PA-CoA entry into ER lumen where it catalyzes the formation of palmitoylcarnitine from PA-CoA.[22] Palmitoylcarnitine is amphipathic and has pleiotropic effects including membrane destabilization, lysis, reduced membrane potential, calcium release and caspase activation.[23], [24] CPT1 relies on L-carnitine as a co-carrier of acyl-CoA and an increased PA load would drain cellular L-carnitine stores resulting in decreased availability of acyl-CoA and thus in a decreased formation of acetylcholine.[25], [26] Limiting the ability of neurons to synthesize acetylcholine would compromise maintenance and trigger neuronal loss. Another important aspect of exhausted neuronal L-carnitine stores is impediment of glucose oxidation resulting in decreased energy production. Depleting the mitochondrial acetylcarnitine levels decreases pyruvate dehydrogenase activity and increases acetyl-CoA levels resulting in attenuation of the Krebs cycle.[26], [27] Reversal of PA-induced neuronal loss by ALA can be explained by its vital role, as a co-enzyme, in the pyruvate- and alpha-ketoglutarate dehydrogenase complexes.[28] In addition to its metabolic role ALA is also a powerful antioxidant,[19] and part of it rescue potential on PA-induced neuronal loss is most likely by ROS scavenging. ROS are important cellular redox regulators, fostering the unfolded protein response (UPR) and ER stress, eventually leading to programmed cell death.[29], [30] In the present study PA-induced neuronal loss was unaltered by presence of the chaperone 4PBA, thus excluding UPR-mediated ER stress. Oxidative stress may still be a culprit, as palmitoylcarnitine and altered phospholipid formation increase ER permeability and ROS discharge.[31], [32]

The protective mechanism of etomoxir on PA-induced neuronal loss is likely explained by its CPT1 inhibition. PA-CoA accumulation is, in contrast to palmitoylcarnitine, not amphipathic, and forms phospholipids and/or other lipid intermediates.[33] Neuronal rescue by exogenously added L-carnitine can be explained by replenishment of available L-carnitine stores.

AMPK activation by the AMP analog AICAR caused myenteric neuronal loss *in vitro*, comparable to that of PA. Simultaneous addition of AICAR and PA did not further augment the neuronal loss, leading to an initial assumption that PA, as shown in a hypothalamic cell line,[34] activated AMPK. This seems unlikely though as ALA and etomoxir, both able to counteract PA-induced neuronal loss, were unable to reverse AICAR- or AICAR plus PA-induced neuronal loss. From this we conclude that AMPK-induced neuronal loss apparently occurs in myenteric neurons. This pathway is, however, not activated by PA and not executed through activation of CPT1.

Damaged neurons display various morphological signs of death. [35], [36] PA exposure enhanced chromatin condensation within cultured myenteric neurons, suggesting programmed cell death. Approximately 18% of all cultured neurons displayed activated caspase-3 immunoreactivity irrespective of treatment (own unpublished observations). Since this finding is not in accord with the observed neuronal loss we question if activated caspase-3 can be considered an optimal marker for apoptosis in myenteric neurons. This conclusion is supported by other studies on peripheral neurons.[11], [13], [37]

### Mechanisms behind OA-induced neuroprotection

OA is, by way of PPAR-alpha activation and upregulated expression of GAP43, reported to protect cultured neurons from PA-induced loss.[16] In the present work we identified a marked neuroprotective capacity of OA *per se* on myenteric neurons; this effect was not attenuated by the presence of PPAR inhibitor. In addition OA significantly counteracted both PA- and AICAR-induced neuronal loss. OA mediated reversal of PA-induced neuronal loss did not involve PPAR activation, while its reversal of AICAR-induced neuronal loss did. This further supports that PA- and AICAR-induced neuronal loss are differently executed. OA's reversal of PA-induced neuronal loss is suggested to involve redirection of PA towards undamaging lipid intermediates, as suggested in other tissues.[38]–[40]

### PA and OA effects on intestinal smooth muscle cells

Striated and vascular muscle cells efficiently take up fatty acids *in vitro*.[39], [41] PA exposure results in apoptosis,[41], [42] ER stress and abnormal lipid distribution that can be counteracted by OA.[39], [41] The beneficial effects of OA have been ascribed to its promotion of triglyceride and lipid droplet formation and decreased formation of phopholipids, resulting in low lipotoxicity.[38], [39] Conflicting data suggests OA to be both apoptotic and anti-apoptotic.[40], [43], [44] Current results showed that OA alone, like PA, reduced survival of intestinal muscle cells. It is important to note that simultaneous treatment with PA and OA failed to execute the apoptotic and anti-proliferative responses obtained with each FA alone. Ceramide- and ROS- formation, ER stress and beta-oxidation as well as AMPK- and PPAR-activation were excluded as causing PA- or OA-induced muscle cell loss. We suggest that the combined PA and OA treatment promoted maintenance of intestinal muscle cells by dual mechanisms; a sustained proliferation and shuttling or redistribution of lipids towards formation of triglycerides. The latter suggestion is based on the finding that simultaneous presence of PA and OA dramatically increased lipid droplet formation in muscle cells. Interestingly, this is well in line with the here reported observation that HFD mice, in contrast to ND mice, harbored numerous lipid droplets in muscularis propria.

### PA effects on glia

Survival of glia *in vitro* was unchanged by PA, OA, ALA, and AICAR treatments. PA notably altered glia morphology. The cytoplasmatic expansion of glia may be in response to PA-induced muscle cell loss, ensuring a supportive layer for the neurons, or rely on the neuronal loss. Astrocytes, closely resembling enteric glia, take on a flat and extended shape when cultured alone, but adapt a stellate shape when co-cultured with neurons.[45] A rounded glia phenotype is suggested to accompany alterations in chloride homeostasis, a process coupled to physiological requirements.[45] Present data does not allow for speculations on altered chloride currents or the consequences thereof, but suggests phenotypic changes to be secondary to PA-induced loss of neurons and/or muscle cells.

### Conclusions

Long term intake of HFD caused a remarkable loss of intestinal myenteric neurons. HFD contains substantial amounts of PA and OA; putative candidates for mediating the HFD-induced neuropathy. As tested *in vitro* PA was capable of mimicking HFD-induced neuronal loss while OA was neuroprotective. PA or OA-exposure both caused intestinal muscle cell loss while glia numbers were unchanged. PA-induced neuronal loss is suggested to be multifaceted. The present data suggests intracellular metabolic derangements due to L-carnitine depletion and palmitoylcarnitine formation to be central. This ultimately results in lack of energy substrates, reduced acetylcholine synthesis, membrane deterioration and oxidative stress. The mechanism leading to PA- or OA-induced loss of intestinal muscle cells is suggested to be unbalanced lipid metabolism, as simultaneous exposure to PA and OA left intestinal muscle cells unaffected in number and with a high intracellular lipid droplet accumulation. The latter probably serving as a reservoir halting lipid metabolism. An increment in intramuscular lipid accumulation, as noted in HFD mice, may explain their well-preserved muscularis propria.

This is the first study showing that long term intake of HFD leads to ENS neuropathy and an imminent GI dysmotility in rodents. Based on the here presented *in vitro* studies we suggest PA as a putative candidate mediating this. Whether these findings can be extrapolated into the clinic needs further investigations.
